# Case Report: Filling Defect in Posterior Semicircular Canal on MRI With Balanced Steady-State Gradient-Echo Sequences After Labyrinthine Ischemia in the Common Cochlear Artery Territory as an Early Sign of Fibrosis

**DOI:** 10.3389/fneur.2020.608838

**Published:** 2021-01-13

**Authors:** Andrea Castellucci, Emanuela Pepponi, Annalisa Bertellini, Caterina Senesi, Margherita Bettini, Cecilia Botti, Salvatore Martellucci, Pasquale Malara, Silvia Delmonte, Francesco Maria Crocetta, Martina Fornaciari, Francesca Lusetti, Giovanni Bianchin, Angelo Ghidini

**Affiliations:** ^1^ENT Unit, Department of Surgery, Azienda USL – IRCCS di Reggio Emilia, Reggio Emilia, Italy; ^2^Department of Radiology, Azienda USL – IRCCS di Reggio Emilia, Reggio Emilia, Italy; ^3^Department of Neurology, Azienda USL – IRCCS di Reggio Emilia, Reggio Emilia, Italy; ^4^Audiology and Ear Surgery Unit, Azienda USL – IRCCS di Reggio Emilia, Reggio Emilia, Italy; ^5^Clinical and Experimental Medicine, University of Modena and Reggio Emilia, Modena, Italy; ^6^ENT Unit, Santa Maria Goretti Hospital, Azienda USL Latina, Latina, Italy; ^7^Audiology and Vestibology Service, Centromedico Bellinzona, Bellinzona, Switzerland

**Keywords:** common cochlear artery, labyrinthine ischemia, posterior semicircular canal, labyrinthine fibrosis, video-head impulse test, vestibular-evoked myogenic potentials, inner ear MRI

## Abstract

We describe a rare case of posterior semicircular canal (PSC) fibrosis following acute labyrinthine ischemia in the territory supplied by the common cochlear artery (CCA) and review the relevant literature. A 71-year-old man with multiple vascular risk factors presented 12 days after the onset of acute vertigo and profound left-sided hearing loss. Right-beating spontaneous nystagmus with downbeat components elicited by mastoid vibrations and headshaking was detected. The video head impulse test (vHIT) revealed an isolated hypofunction of the left PSC, whereas vestibular evoked myogenic potentials (VEMPs) showed ipsilateral saccular loss. The clinical presentation and instrumental picture were consistent with acute ischemia in the territory supplied by left CCA. Compared to previous imaging, a new MRI of the brain with 3D-FIESTA sequences highlighted a filling defect in the left PSC, consistent with fibrosis. Hearing function exhibited mild improvement after steroid therapy and hyperbaric oxygen sessions, whereas vHIT abnormalities persisted over time. To the best of our knowledge, this is the only case in the literature reporting a filling defect on MRI, consistent with semicircular canal fibrosis following acute labyrinthine ischemia. Moreover, PSC fibrosis was related with poor functional outcome. We therefore suggest using balanced steady-state gradient-echo sequences a few weeks following an acute lesion of inner ear sensors to detect signal loss within membranous labyrinth consistent with post-ischemic fibrosis. Besides addressing the underlying etiology, signal loss might also offer clues on the functional behavior of the involved sensor over time. In cases of acute loss of inner ear function, a careful bedside examination supplemented by instrumental assessments, including vHIT and VEMPs, of vestibular receptors and afferents may be completed by MRI with balanced steady-state gradient-echo sequences at a later time to confirm the diagnosis and address both etiology and functional outcome.

## Introduction

Inner ear fibrosis and ossification result from fibrous tissue deposits and new bone formation, respectively, in labyrinthine structures. These conditions represent subsequent final steps of several inner ear pathologies, including infections, genetic and autoimmune diseases, trauma, and ischemia (1). Animal studies on labyrinthine arterial obstruction have demonstrated that ischemia may result in end-organ fibrosis within 2 weeks and ossification within a few months (2, 3). Similarly, inner ear fibrosis has been observed in post-mortem examination of patients who had suffered an acute labyrinthine ischemia (4, 5).

Magnetic resonance imaging (MRI) with balanced steady-state gradient-echo sequences has demonstrated higher sensitivity in detecting signs of early fibrosis compared to high-resolution CT (HRCT) scans (6). In particular, steady-state gradient-echo MRI such as FIESTA (fast imaging employing steady-state acquisition) and constructive interference in steady state (CISS) sequences with multiplanar reconstructions can offer a high-contrast evaluation of the signal loss within the fluid-filled spaces on the membranous labyrinth due to fibrotic tissue deposits (7).

The development of modern tools for vestibular testing, such as video head impulse test (vHIT) and vestibular evoked myogenic potentials (VEMPs), has allowed fast measurements of the activity of each semicircular canal (SC) and otolith receptors/afferents in clinical settings (8, 9). Thanks to the interpretation of data obtained from matching results from assessments of all five inner ear sensors, it has become possible to identify specific lesion patterns and offer reliable hypothesis on underlying etiopathological mechanisms affecting inner ear receptors or vestibular nerve branches.

Here we describe the onset of posterior semicircular canal (PSC) fibrosis on MRI in a patient with a clinical presentation and instrumental findings consistent with acute stroke in the territory supplied by the common cochlear artery (CCA). We also review the relevant literature.

## Case Description

**Figure 1:**
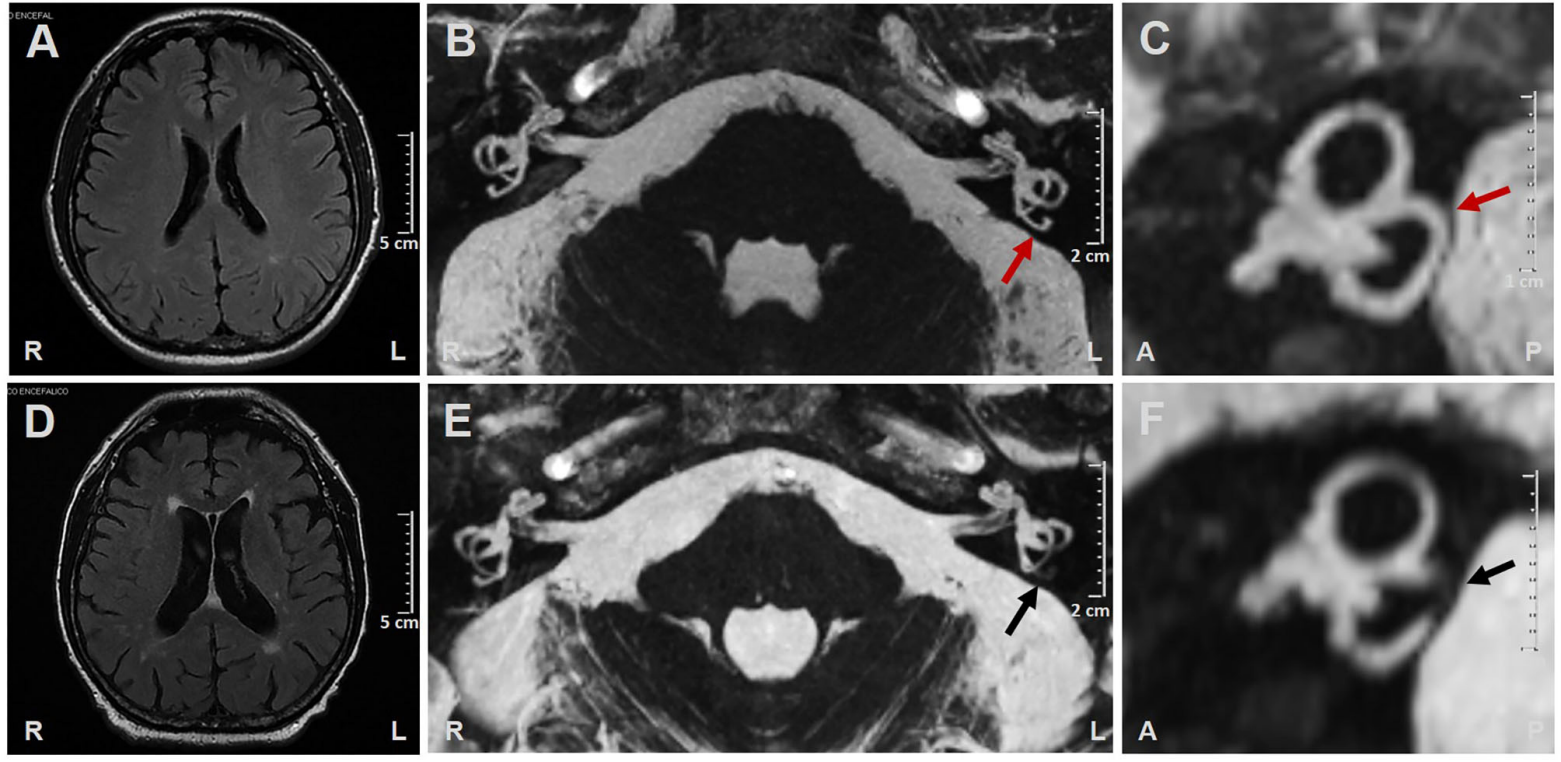
**(A–C)** Brain MRI performed 4 years prior to left-sided labyrinthine ischemia exhibiting **(A)** Slight signs of periventricular leukoaraiosis on axial T2-weighted FLAIR (fluid-attenuated inversion recovery) images. **(B)** Axial and **(C)** sagittal MRI with 3D-FIESTA (fast imaging employing steady-state acquisition) sequences showing normally fluid filled semicircular canals on both sides (red arrows indicate left posterior semicircular canal). **(D–F)** Brain MRI performed 14 days after left-sided labyrinthine ischemia showing **(D)** Increased areas of periventricular leukoaraiosis and widened cerebrospinal fluid spaces on atrophic basis on axial T2-weighted FLAIR images. **(E)** Axial and **(F)** sagittal MRI with 3D-FIESTA sequences showing filling defect within the left posterior semicircular canal (black arrows). A, anterior; L, left; P, posterior; R, right.

A 71-year-old man was admitted to our institution for evaluation of sudden onset of left-sided hearing loss (HL), vertigo, and severe unsteadiness persisting for over 12 days. His clinical history was consistent with arterial hypertension, atrial fibrillation (in treatment with oral anticoagulants), and myelodysplastic syndrome (regular hematologic follow-ups). He reported a transient ischemic attack 4 years earlier but denied any previous inner ear symptoms. Corresponding brain MRI images were reviewed, showing signs of periventricular leukoaraiosis and normal posterior fossa structures, including normally fluid-filled SCs on FIESTA sequences (Figures 1A–C).

**Figure 2:**
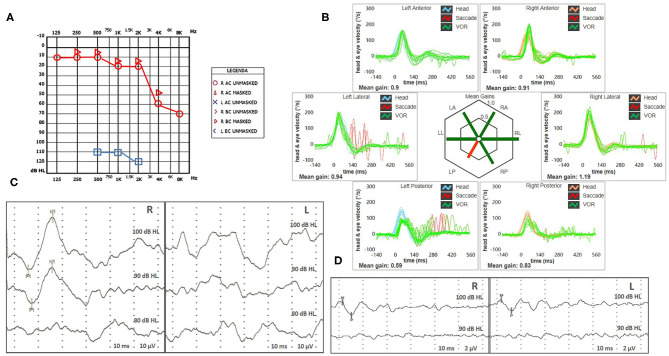
**(A,B)** Presenting scenario including **(A)** Pure-tone audiometry exhibiting right-sided high-frequency sensorineural hearing impairment and profound left-sided hearing loss. **(B)** vHIT. Blue lines represent head impulses exciting left canals, orange lines correspond to impulses for right canals, green lines represent eye movements induced by the activation of VOR following each impulse and red lines correspond to corrective saccades. Mean value of VOR-gain (eye velocity/head velocity) is reported for each canal. The hexagonal plot in the center of the figure summarizes mean VOR-gains for each canal; normal gains are shown in green and deficient gains are in red. A selective deficient VOR-gain for the left posterior semicircular canal (0.59) with overt saccades can be observed. Cervical VEMPs **(C)** and ocular VEMPs **(D)** for air-conducted sounds. For cervical VEMPs, right and left lines correspond to myogenic responses (p1–n1) recorded on the right and left SCM muscle (i.e., right and left saccular responses), respectively. For ocular VEMPs, being crossed responses, right and left lines represent potentials (n–p) recorded under the left and right eye (i.e., right and left utricular responses), respectively. VEMPs testing revealed normal responses on the right side (83 μV at 100 dB HL stimuli) and absent potentials on the left, whereas symmetrical amplitudes for ocular VEMPs (R: 6 μV and L: 5 μV at 100 dB HL stimuli) could be detected. L, left; LA, left anterior; LL, left lateral; LP, left posterior; R, right; RA, right anterior; RL, right lateral; RP, right posterior; SCM, sternocleidomastoid; vHIT, video head impulse test; VEMPs, vestibular evoked myogenic potentials; VOR, vestibulo-ocular reflex.

Vestibular examination with video Frenzel goggles detected sustained spontaneous right-beating nystagmus exhibiting downbeat/right-torsional components after mastoid vibrations and headshaking. Oculomotor testing and a neurological examination ruled out central nervous system involvement, as did a brain CT scan. Tympanic membranes were unremarkable on micro-otoscopy. Pure-tone audiometry showed right-sided high-frequency sensorineural HL consistent with the patient's age and profound left-sided hearing impairment (Figure 2A). An ICS Impulse device (Otometrics, Natus Medical Inc, Denmark) was used to measure the vestibulo-ocular reflex (VOR) gain for all six semicircular canals. Gains were considered normal if >0.8 for lateral canals and >0.7 for vertical canals (8). Selective mild reduction of left posterior SC (PSC) activity was detected on vHIT (Figure 2B). A 2-channel evoked potential acquisition system (Viking, Nicolet EDX, CareFusion, Germany) was used for cervical and ocular VEMPs testing. Potentials were measured by delivering tone bursts (frequency: 500 Hz, duration: 8 ms, stimulation rate 5 Hz) *via* headphones. The recording system used an EMG-based biofeedback monitoring method to minimize variations in muscle contractions and VEMPs amplitudes. Each stimulus was retested to assess reproducibility of responses, and the stimulus intensity was reduced by 10 dB until the threshold for each side was reached. Although no cervical responses could be detected on the pathologic side consistent with left-sided isolated saccular loss, symmetrical potentials were evoked for ocular testing (Figures 2C,D). Clinical presentation and instrumental findings suggested a labyrinthine ischemia in the territory supplied by the left CCA.

The patient immediately began steroid therapy (1 week intravenous 1 mg/Kg dexamethasone followed by oral tapering for 1 additional week) and oral treatment with 48 mg/day betahistine for 2 weeks, in accordance with recommendations available in the literature (10). Simultaneously, he received 15 sessions of hyperbaric oxygen therapy, and a cardiologic evaluation with transcranial Doppler assessment was requested to adjust anticoagulant and antihypertensive therapy, if necessary. Since stenosis or blood flow abnormalities within the main intracranial arteries were excluded, current therapy was continued.

Due to related cardiovascular risk factors, a new brain MRI was also scheduled in the following days. Acute brainstem infarction in diffusion-weighted and T2-weighted fluid-attenuated inversion recovery (FLAIR) images were excluded, whereas widened cerebrospinal-fluid spaces on atrophic basis and increased areas of leukoaraiosis compared with previous neuroimaging were detected (Figure 1D). 3D-FIESTA sequences detected signal loss within left-sided PSC consistent with fibrous tissue deposits (Figures 1E,F).

**Figure 3:**
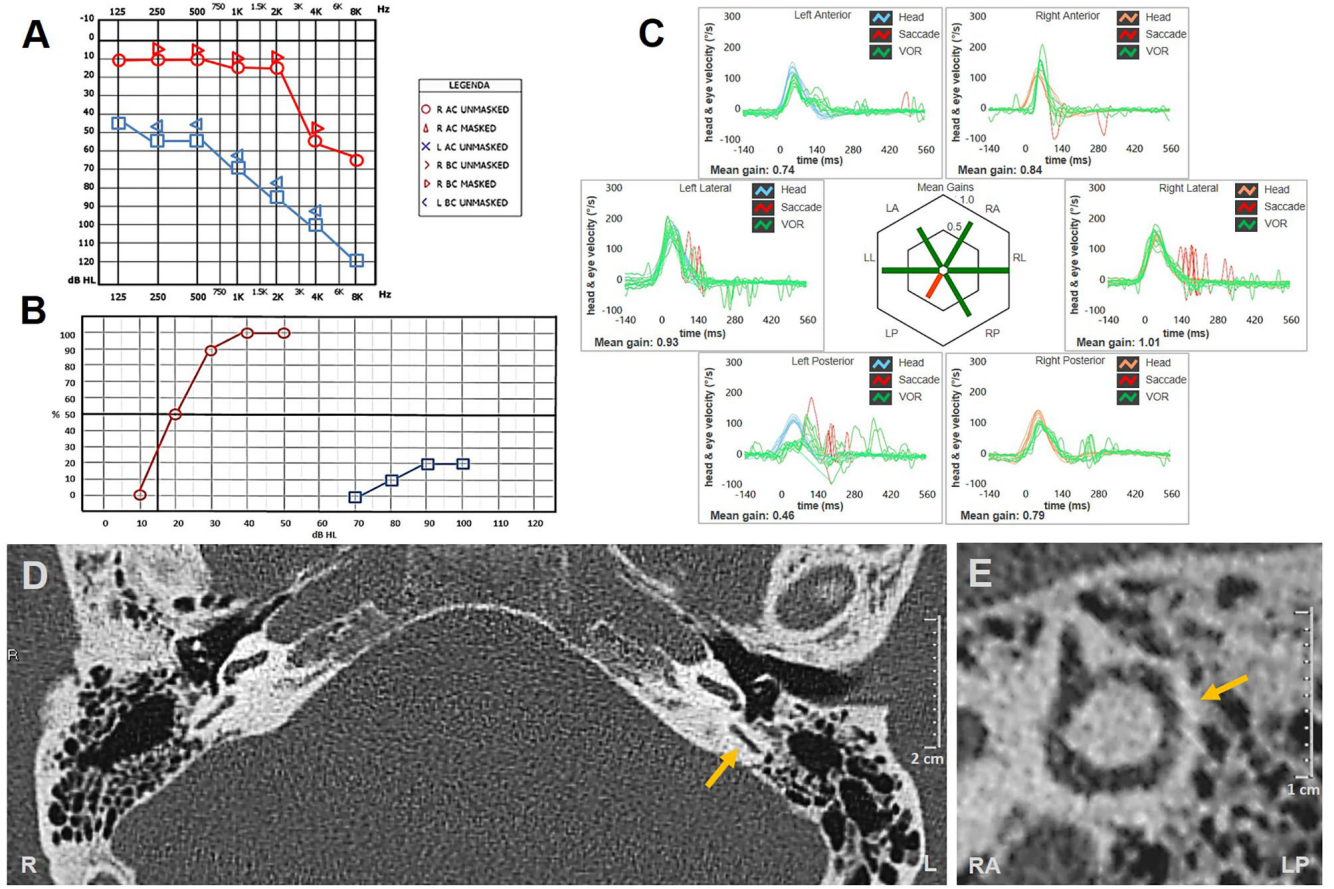
**(A–C)** Instrumental picture following steroids and hyperbaric oxygen therapy. **(A)** Pure-tone audiometry exhibiting partial recovery for right-side hearing function with a pure-tone average (500 Hz−4 kHz) of 77.5 dB. **(B)** Standard speech audiometry in silent setting showing optimal (100%) and poor (20%) speech discrimination score on the right and left sides, respectively. **(C)** vHIT showing persistent selective loss for left posterior canal VOR-gain (0.46) with both overt and covert saccades. Affected canal VOR-gain is further impaired compared to presenting values. **(D,E)** Temporal bones HRCT scans with axial **(D)** and parasagittal reconstructed image along the Stenver plane **(E)** excluding signs of posterior semicircular canal ossification on the left side (yellow arrows). HRCT, high-resolution computed tomography; L, left; LA, left anterior; LL, left lateral; LP, left posterior; R, right; RA, right anterior; RL, right lateral; RP, right posterior; vHIT, video head impulse test; VOR, vestibulo-ocular reflex.

At 3-week evaluation with video Frenzel goggles, slight spontaneous right-beating nystagmus enhanced by mastoid vibrations and headshaking could still be detected. Hearing evaluation with pure-tone audiometry showed a moderate to severe down-sloping sensorineural HL on the left side, consistent with mild recovery of cochlear function (Figure 3A). Nevertheless, word recognition on the left side was poor on speech audiometry, while the speech discrimination score was optimal on the healthy side (Figure 3B). However, the patient refused additional therapy with intratympanic steroids.

Although vHIT showed further reduction in left-sided PSC activity (Figure 3C), the patient reported that his vestibular symptoms were substantially relieved. Rehabilitation was therefore not pursued, and the patient refused to undergo additional testing, including VEMPs reassessment and caloric testing. Temporal bone HRCT performed 2 weeks later finally excluded signs of labyrinthine ossifications (Figures 3D,E).

Written informed consent was obtained from the patient for the publication of this case report, including all data and images.

## Discussion

**Figure 4:**
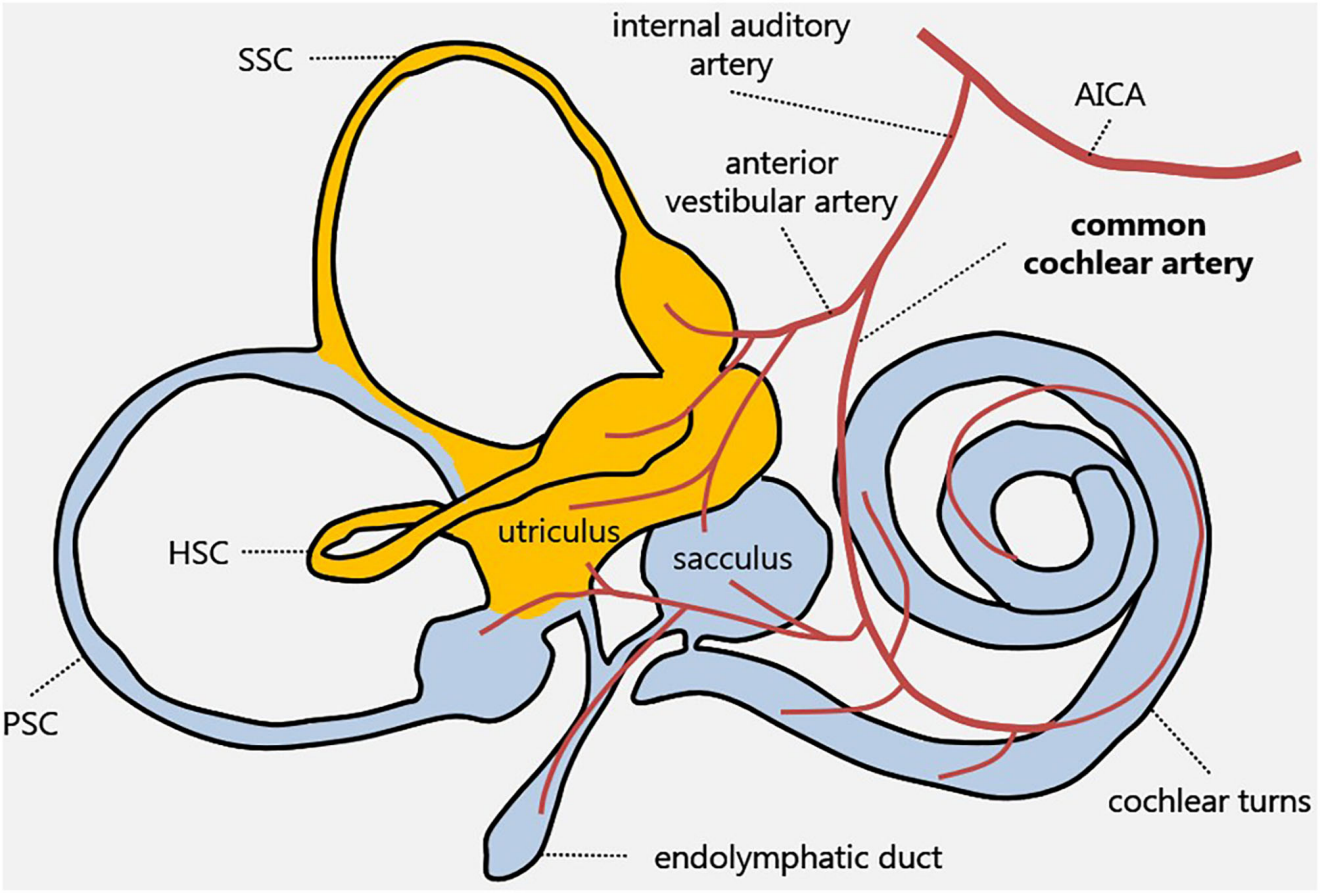
Inner ear vascular supply. Labyrinthine receptors mainly supplied by the anterior vestibular artery and the common cochlear artery are in yellow and in light blue, respectively. AICA, anterior-inferior cerebellar artery; HSC, horizontal semicircular canal; PSC, posterior semicircular canal; SSC, superior semicircular canal.

The inner ear is supplied by the internal auditory artery, which branches from the anterior-inferior cerebellar artery (AICA) and divides into two main terminal branches: the anterior vestibular artery and CCA. Whereas, the first mostly supplies the utricle and the horizontal and superior SCs, the latter mainly serves the cochlear turns, saccule, and PSC (11, 12) (Figure 4).

In recent years, thanks to the introduction in clinical practice of fast modern devices such as vHIT and VEMPs, which provide a precise assessment for each SC and the otolith end organs in the high-frequency domain, instrumental testing has demonstrated its pivotal role in the topographical diagnosis of selective dysfunction of inner ear structures, including in the acute stage (8, 9). Nevertheless, underlying etiologic mechanisms may remain unclear. In fact, whereas ischemic damage should always be suspected when symptoms consistent with peripheral vestibular loss are accompanied by sudden HL, in cases of isolated vertigo of peripheral origin, clinical and instrumental assessments may not clarify whether an inflammatory or vascular lesion represents the underlying mechanism (13–16). For example, a partial vestibular hypofunction sparing saccular and PSC activity may be due either to neuritis involving the superior nerve division or to anterior vestibular artery stroke (15, 17). Conversely, a selective ischemia in the territory supplied by CCA seems to represent the most likely disorder accounting for an acute functional loss of cochlear epithelium, saccular macula and PSC ampulla (13, 18, 19). The same lesion pattern was detected in our patient exhibiting profound left-sided HL, abnormal cervical VEMPs on the same side and selective VOR-gain reduction for ipsilateral PSC. Furthermore, asymmetrical stimulation of vertical SCs resulting from an acute selective loss for left-sided PSC activity may likely account for right-torsional/downbeat components elicited by mastoid vibrations and headshaking in the acute stage (20, 21). On the other hand, different levels of vestibular compensation or hypothetical involvement of hair cells encoding low-frequency stimuli for left horizontal SC may explain horizontal paretic nystagmus detected at the follow-up evaluation. Unfortunately, the patient refused to undergo the caloric test, which assesses horizontal VOR in the low-frequency domain, so we could not test type II hair cells within the horizontal SC and regular afferents running along the superior division of the vestibular nerve.

Steady-state gradient-echo MRI such as FIESTA and CISS sequences offer a high signal-to-noise ratio that allows submillimeter imaging and high-quality multiplanar reconstructions. These images are widely used when a high-contrast evaluation of the fluid-filled spaces on the membranous labyrinth is required to check for cochlear patency and inner ear malformations in patients scheduled for cochlear implant surgery (22, 23). The same algorithms are generally included in MRI protocols to evaluate the internal auditory canal content for VIII cranial nerve tumors due to the high contrast between fluids and solid structures (24–26). Conversely, in cases of suspected posterior fossa stroke, other sequences, such as diffusion-weighted MRI, are routinely used to rule out acute infarct lesions (27). Nevertheless, an accurate bedside examination with a 3-step bedside oculomotor testing called the HINTS protocol (head impulse, observation of nystagmus in different gaze positions and test of skew) has demonstrated its ability to detect an acute stroke of the posterior fossa within the first 48 h with higher sensitivity compared to early MRI (27). More recently, further studies have concluded that including hearing evaluation in the protocol (HINTS plus) can add a substantial contribution to detecting AICA strokes (28). Therefore, patients presenting with acute cochleovestibular symptoms and high vascular risk factors should always be scheduled for a brain MRI at 48 h to rule out associated brainstem infarction. Conversely, in cases where symptoms are likely due to peripheral disorders, bedside examination complemented by instrumental inner ear assessment should adequately guide the detection of the lesion site; neuroimaging can be postponed to rule out VIII cranial nerve lesions and/or labyrinthine abnormalities.

To the best of our knowledge, this is the first report showing newly formed inner ear fibrosis based on MRI findings before and after a CCA ischemic lesion. Besides clinical and instrumental findings supporting this etiopathogenetic hypothesis, labyrinthine ischemia is also consistent with the high cardiovascular risk factors exhibited by the patient. Additional MRI data, including pronounced periventricular leukoaraiosis and wide cerebrospinal fluid spaces, are also in accordance with atrophic sequelae following chronic hypoxic insult to the brain tissue. The aforementioned findings strengthen the assumed mechanism underlying the presenting scenario involving ischemic mechanisms. It might be postulated that the lack of concurrent cochlear and saccular abnormalities on imaging may reflect possible variable susceptibility to steroid and oxygen therapy among different hair cells or different inter-individual patterns of venous drainage among inferior labyrinthine structures, making PSC more vulnerable to hypoxia in this case (11). The asymmetrical extent of damage among inner ear receptors sharing the same vascular supply from CCA can likely account for the different behavior exhibited by each sensor over time. In fact, whereas cochlear function mildly improved following treatment, though not achieving a serviceable hearing level, left-sided PSC activity was found further impaired on follow-up. This aspect seems to be in agreement with recent studies correlating loss of vestibular function with filling defect within the inner ear on MRI (6, 23). Unfortunately, VEMPs could not be reassessed to check whether saccular function recovered as expected in accordance with the lack of corresponding radiological abnormalities, despite the fact that patients exhibiting chronic white matter lesions on MRI seem to develop worse VEMPs outcomes after acute vestibular loss (29).

Temporal bone HRCT scans performed almost 2 months after symptom onset showed normally patent inner ear structures, excluding progression of damage toward ossification. Nevertheless, due to the short-term follow-up, we cannot exclude a possible late onset of PSC ossification in our patient.

Since balanced steady-state gradient echo sequences represent a reliable tool to detect inner ear fibrosis irrespective of the patients' age (7, 30), it seems reasonable to recommend considering CCA ischemia in cases of filling defects for PSC in patients with acute onset of HL and vertigo. Clinicians should be aware of the eventuality of a labyrinthine stroke, as it has been demonstrated how peripheral ischemic lesions may precede a major stroke involving posterior fossa structures (31, 32). Furthermore, as already suggested, clinicians are encouraged to seek causes of dizziness other than the sole chronic white matter lesions, given that small vessel disease is often associated to other peripheral vestibular disorders, as has already been described (33).

Though this report likely represents a unique case with obvious limitations related to single-case reports without histopathological support, it might be assumed that post-ischemic fibrosis of inner ear receptors could not represent an exceptional finding, particularly when we consider that steady-state gradient-echo MRI sequences are not routinely included in the imaging protocol adopted for acute HL and vertigo. Therefore, in the case of acute cochleovestibular symptoms where a careful clinical/instrumental assessment orients the diagnosis toward a peripheral lesion, along with recommending a global vestibular assessment with vHIT and VEMPs, we would also suggest postponing neuroimaging and routinely using the 3D-FIESTA MRI sequences to detect possible signs of post-ischemic fibrosis. This finding may be extremely helpful especially in those cases where underlying etiopathological mechanisms are not adequately addressed by clinical and instrumental assessments, which was not the case for our patient.

## Conclusion

The patient described in this report exhibited on MRI an unusual filling defect consistent with fibrosis in the PSC following acute labyrinthine ischemia. This finding seems also to be related to poor functional outcome for the affected end organ. Therefore, it may be reasonable to suggest using balanced steady-state gradient-echo sequences a few weeks following an acute lesion of the inner ear sensors, as they might detect signal loss within the membranous labyrinth suggesting post-ischemic fibrosis. This finding, besides addressing the diagnosis in cases where the underlying etiopathological mechanisms are unclear, might hypothetically provide clues on the functional behavior of the involved inner ear sensors over time. However, additional reports reproducing similar findings with longer follow-up are needed before any conclusion can be reached on the clinical value of filling defects within inner ear on MRI in patients with acute cochleovestibular symptoms.

## Data Availability Statement

The raw data supporting the conclusions of this article will be made available by the authors, without undue reservation.

## Ethics Statement

Written informed consent was obtained from the patient for the publication of any potentially identifiable images or data included in this article.

## Author Contributions

AC, EP, and AB: conceptualization, investigation, data acquisition and interpretation, and original draft preparation. AC: images and artwork. CS, MB, CB, SD, FC, MF, and FL: investigation, data collection, and original draft preparation. SM and PM: substantial role in data interpretation and manuscript revision. GB and AG: supervision and manuscript review. All authors approved the final version of the manuscript.

## Conflict of Interest

The authors declare that the research was conducted in the absence of any commercial or financial relationships that could be construed as a potential conflict of interest.
